# Medical Miscellany

**Published:** 1854-02

**Authors:** 


Medical Miscellany.—Have a care regarding the stuff sold at the shops for "fly poison" and called cobalt. It is no cobalt, but arsenic and nothing else.—An infant, near Boston, only two weeks old, has a set of perfectly formed teeth.——On sawing open a locust log, at Poughkeepsie, N. Y. thought to be a hundred years old, a live toad weighing seven and a half pounds, was found in the center.——Dr. Bull, one of the most distinguished surgeons of Cork, Ireland, committed suicide whilst laboring under temporary mental aberation.——Arago, the illustrious Astronomer and Meteorologist, and perpetual Secretary of the Institute of France, died on the 2d of October, at the age of 67 years.——A new petroleum, or oil spring, has recently been discovered in Virginia—it furnishes some two gallons of oil, a day.——The United States has raised $1600 towards the Jenner Monument—a larger sum than any other country has subscribed.—A New York surgeon states that he has tied the carotid artery thirty-six times.——During the month of July, Aug. and Sept., there were, in the city of N. York 5077 births and 7111 deaths, an excess of deaths, amounting to 2034.——The cholera is raging in Europe and seems to be threatening this country, and may be expected next spring, unless prevented by judicious measures.——Dr. J. V. C. Smith, Editor Boston Med. and Surgical Journal, has been elected Mayor of Boston——Dr. Waters, of Pittsburgh, removed a worm about one-eight of an inch long, from the right eye of a patient; it had lately grown rapidly; it obstructed vision and caused severe pain; was of a whitish color and very lively,——A bit of smoked herring is said to destroy the nauseous taste of cod liver oil; chew it before and after taking the oil.——Dr. Carpenter, the celebrated Physiologist, has issued a needed and popular work, on the physiological effects of the use of alcoholic poisons, upon the human system. ——A new and spacious hospital is to be erected at Albany, over $50,000 having been subscribed; to be under the auspices of Albany Medical College.——When Gen. Washington was in New York, soon after the revolution, he had a set of false teeth inserted by the only dentist in the city; now there are nearly one thousand dentists there.——It is stated that the hippopotamus who swallowed a lady's lap dog lately, at the public gardens, did so under the impression that he was taking a dose of *bark*.——During the most prominent year of the Salem Witchcraft, 1692, the number of executioners was twenty, sentences of death eight and 150 persons imprisoned in jail.——Whales are often 120 feet long, and weigh from fifteen to twenty tons.

